# Detection of potential transmission foci of lymphatic filariasis using molecular xenomonitoring in Huahine, French Polynesia

**DOI:** 10.1371/journal.pntd.0013492

**Published:** 2025-09-19

**Authors:** Reva Lannuzel, Tanagra Lambert, Farah Deen, Hmeniko Tourancheau, Jérôme Marie, Michel A. Cheong Sang, Manfred Mervin, Benoit Stoll, Hervé C. Bossin, Françoise Mathieu-Daudé

**Affiliations:** 1 Medical Entomology Laboratory, Institut Louis Malardé (ILM), Ifremer, IRD, UPF, UMR SECOPOL, Tahiti, French Polynesia; 2 Institut de Recherche pour le Développement (IRD), ILM, Ifremer, UPF, UMR SECOPOL, Tahiti, French Polynesia; 3 UMR MIVEGEC, Univ. Montpellier, IRD, CNRS, Tahiti, French Polynesia; 4 University of French Polynesia (UPF), ILM, Ifremer, IRD, UMR SECOPOL, Tahiti, French Polynesia; Cyprus International University: Uluslararasi Kibris Universitesi, CYPRUS

## Abstract

**Background:**

In French Polynesia, substantial progress has been achieved in eliminating lymphatic filariasis (LF) caused by *Wuchereria bancrofti* var. *pacifica*, a parasite transmitted by the mosquito vector *Aedes polynesiensis*. However, despite multiple rounds of Mass Drug Administration (MDA), LF transmission persists on some islands, underscoring the need for robust surveillance to evaluate transmission risks and identify potential transmission foci.

**Methodology/principal findings:**

An extensive entomological survey combined with a Molecular Xenomonitoring (MX) study was conducted on Huahine Island in the Leeward Islands (Society Islands), where new LF cases continue to be reported. Adult mosquitoes were collected from 420 sampling points across 28 Primary Sampling Units (PSUs) to map mosquito species distribution and estimate infection prevalence in mosquitoes. Among the 5508 female mosquitoes collected, *Ae. polynesiensis* was the predominant species (74%), widely distributed across the island and particularly abundant in some PSUs. Other species included *Aedes aegypti* (20%) and *Culex quinquefasciatus* (4%). Mosquito pools from species of the genera *Aedes* and *Culex* were tested for the presence of *W. bancrofti* using real-time PCR. Positive pools were detected in 13 PSUs, involving both vector and non-vector *Aedes s*pecies, *Ae. polynesiensis* (63.6%) and *Ae. aegypti* (36.4%). Estimated infection prevalence in mosquitoes was higher in *Ae. aegypti* (1.1%) than in *Ae. polynesiensis* (0.53%), likely reflecting the differences in species abundance and host preferences. Several potential transmission foci were identified, primarily concentrated in the northern part of the island.

**Conclusions/significance:**

Our study demonstrates the effectiveness of MX using female *Aedes* mosquitoes in identifying potential transmission foci and detecting the presence of LF cases in the vicinity on the island of Huahine. This approach constitutes a valuable tool for post-MDA surveillance in Pacific Islands, where *Aedes* mosquitoes are key vectors for *W. bancrofti*, and will effectively inform the targeted implementation of control interventions, including innovative vector control strategies.

## Author summary

Lymphatic filariasis (LF) is a human disease caused by infection with filarial worms transmitted through mosquito bites. Over recent decades, significant progress has been made toward LF elimination, including in the Pacific islands, thanks to the Global Programme to Eliminate Lymphatic Filariasis (GPELF). However, residual areas of transmission persist in this region, requiring the implementation of effective detection methods. LF infection can be monitored either directly in human populations or indirectly through Molecular Xenomonitoring (MX), which detects the pathogen’s DNA in blood-feeding mosquitoes. Infected mosquitoes represent an indirect proxy for ongoing transmission nearby and for the recent presence of human cases due to their short flight range and limited lifetime. Our study focused on a mosquito-based investigation conducted on the island of Huahine, in French Polynesia. The MX survey identified several potential foci of LF transmission at the district level on the main island. These findings underscore the relevance of MX for monitoring LF elimination efforts in French Polynesia and highlight the need for continued surveillance to sustain progress and guide targeted vector control interventions.

## Introduction

Lymphatic filariasis (LF), a tropical mosquito-borne disease caused by *Wuchereria bancrofti, Brugia malayi* and *Brugia timori* species of nematodes, can result in severe morbidity and disability, such as lymphedema, elephantiasis, or hydroceles [1,2]. This disease was estimated to directly affect around 51 million people in 2018 [2,3]. The World Health Organization (WHO) initiated in 2000 the Global Programme to Eliminate Lymphatic Filariasis (GPELF), a strategy based on annual cycles of Mass Drug Administration (MDA) with ivermectin or diethylcarbamazine in combination with albendazole [1].

In the Pacific region, the Pacific Programme to Eliminate Lymphatic Filariasis (PacELF) was launched in 1999 by the alliance of 22 Pacific Island countries and territories (PICTs), with the goal to achieve global elimination of LF by 2020 [4–6]. To date, monitoring and evaluation of MDA programmes has relied on the assessment of filariasis prevalence in the human population through blood sampling, including antigen detection methodologies such as the Alere Filariasis Test Strip (FTS), which detects *W. bancrofti* antigens [7,8]. Currently, WHO recommends post-MDA human surveillance using Transmission Assessment Surveys (TAS), a standardized evaluation method based on the measurement of LF antigen prevalence in children aged 6–7 years, commonly using rapid diagnostic tools such as filarial immunochromatographic test (ICT card) [9–11]. Seroprevalence values of <1% in areas where *Aedes* species are vectors, or <2% in areas where *Culex* or *Anopheles* are vectors, are used to determine whether transmission has been interrupted [12,13].

Mosquito field sampling and dissection has traditionally been the gold standard for measuring infection rates and densities of parasites in the vector [14,15]. However, conducing an adequate number of dissections becomes increasingly costly, time-consuming and laborious in areas where infection prevalence drops below 1% [11].

Thanks to recent technological advances, Molecular Xenomonitoring (MX), which utilizes polymerase chain reaction (PCR) to detect parasite DNA in mosquito vectors, has proven a valuable tool to evaluate the status of LF transmission [16]. Indeed, MX allows the detection of PCR-positive mosquito pools, providing a proxy for the current or recent presence of infected humans nearby, in relation to the short life span of mosquitoes [10,17]. This technique is more sensitive and less time-consuming than mosquito dissection and less intrusive than TAS for detecting evidence of circulating *W. bancrofti* when prevalence is low [17–19].

Studies have reported the ability of PCR to detect as few as one microfilaria in pools of 50–100 mosquitoes [17,20–22]. When lymphatic filariasis (LF) infection levels become undetectable using antigen (Ag) or microfilariae (Mf) tests, xenomonitoring can complement Transmission Assessment Surveys (TAS) to validate the cessation of mass drug administration (MDA) and monitor for potential LF resurgence [19]. However, challenges remain. There are limited guidelines on sampling strategies, and no clear thresholds have been established for DNA prevalence in mosquitoes that would indicate ongoing transmission [17]. Provisional vector infection thresholds of <0.25% for *Culex*, < 1% for *Anopheles*, and <0.1% for *Aedes* have nonetheless been proposed to guide decisions on stopping MDA [23].

MX has been applied to different species of mosquito vectors of LF belonging to *Aedes, Anopheles* and *Culex*, in several countries, including Samoa [10,17,24,25], Brazil [26], Sri Lanka [18], Egypt [11], Bangladesh [16], India [19,27], Tanzania [28], Togo [29], Ghana [14], and Sierra Leone [30]. It has been used at various stages of monitoring, both pre- and post-MDA, for different objectives, including evaluation of the impact of MDA, assessing its sensitivity in detecting residual transmission foci relative to human seroprevalence surveys, and informing threshold determination for the interruption of transmission [10,16,17,19]. In contexts such as assessing the impact of MDA or supporting decisions to stop interventions, standardized mosquito collection protocols could ensure representative sampling and comparability across time and sites. However, in post-validation surveillance (PVS) settings, where the goal is to detect potential recrudescence, opportunistic or targeted sampling strategies may be operationally preferable and sufficiently informative, particularly when combined with strong entomological and molecular diagnostic capacity.

LF is endemic in French Polynesia [31], and elimination programmes have been implemented under PacELF and GPELF since 1999, using prolonged mass administration of antifilarial drugs targeting *W. bancrofti* to prevent filarial transmission [6]. While LF prevalence has dropped below 1% in most evaluation units, lymphatic filariasis transmission persists in several islands of French Polynesia, necessitating continued elimination efforts in the Society Islands (Leeward Islands, including Huahine), the Southern Marquesas Islands, and the Gambier Archipelago. Despite years of MDA, the prevalence of *Wuchereria* antigenemia remains surprisingly high in Huahine. In 2020, out of 199 people tested in 10 districts on this island, 10.6% were positive [31]. During the Directly Observed Treatment campaigns in 2020 and 2021, which used diethylcarbamazine and albendazole, coverage rates of 91% (2020) and 92% (2021) in Huahine, and 70% and 83%, respectively, in the Southern Marquesas, were achieved among the population [32,33].

In French Polynesia, LF is transmitted by the day-biting *Aedes polynesiensis* (Marks, 1951) [34,35]. The bio-ecology of this mosquito, particularly its use of a wide variety of natural and artificial breeding sites, has made conventional larval control measures less practical. As a result, MDA has historically been the most viable strategy for controlling LF transmission. However, innovative approaches such as the release of male mosquitoes infected with the *Wolbachia* bacteria, known as the Incompatible Insect Technique (IIT), have been employed in French Polynesia, notably on the islet of Tetiaroa, leading to a drastic reduction in the local mosquito populations [36]. Additionally, other “rear & release” techniques, such as the Sterile Insect Technique (SIT) are currently under evaluation in the Pacific region which could provide supplemental tools toward island-wide mosquito vector control [37].

This study aimed to characterize the spatial distribution of LF mosquito vectors and identify potential transmission foci on the island of Huahine (French Polynesia), where human LF cases continue to be reported. By combining an entomological investigation and a MX survey, we sought to generate an entomological risk map and estimate ongoing transmission to support evidence-based decision-making in the context of LF elimination efforts. A secondary objective was to evaluate the feasibility of implementing follow-up MX surveys under operational conditions, specifically assessing whether simplified field-based mosquito identification at the genus level could be sufficient for surveillance, thereby reducing logistical and technical constraint.

## Materials and methods

### Study site and climate

**Fig 1:**
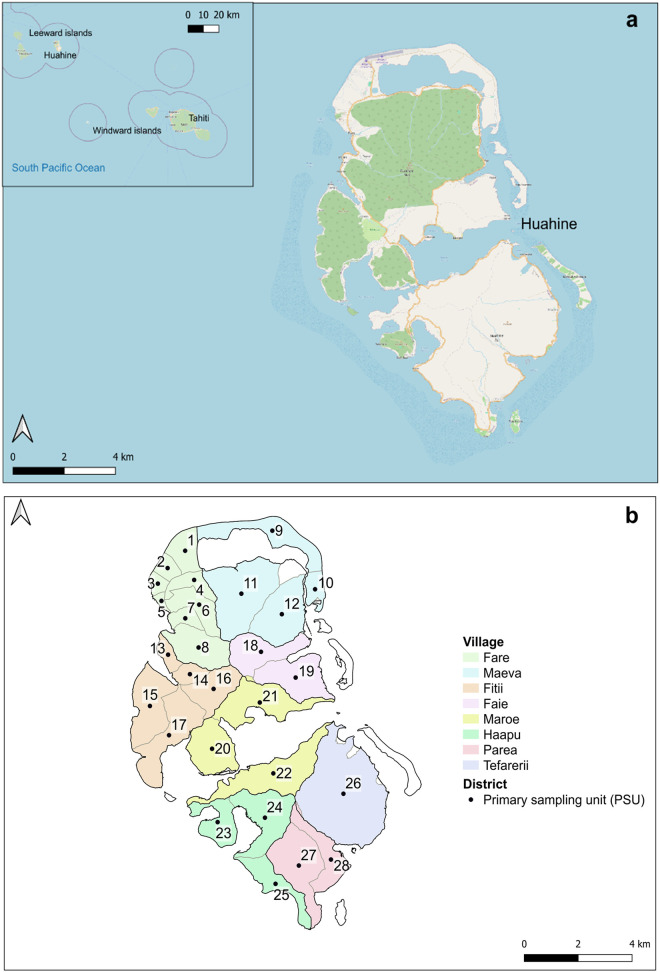
Maps of the study island. (a) Location of Huahine island (in the Leeward islands) relative to Tahiti, the main island of French Polynesia; (b) Map of Huahine (75 km²) showing the 8 villages, each distinguished by a different colour, and the 28 Primary Sampling Units (PSUs) under study, each marked by a dark dot and assigned a unique identification number (PSU code). District boundaries were sourced from the Statistics Institute of French Polynesia. The base map is sourced from OpenStreetMap contributors (https://www.openstreetmap.org/#map=12/-16.7501/-150.9806), available under the Open Database License (ODbL). Maps were generated using QGIS (version 3.30.0, 2023).

The island of Huahine (75 km^2^), located about 170 kilometres northwest of Tahiti (16°4’S, 151°0’W) is part of the Leeward Islands (Society Archipelago, French Polynesia, South Pacific) (Fig 1a). The overall island population, estimated at 6075 inhabitants according to the 2017 population census is distributed across 8 villages: Fare (the main village and administrative centre), Maeva, Faie, Fitii, Parea, Tefarerii, Haapu and Maroe (Fig 1b) [38]. Each village is divided into districts for a total of 34 districts encompassing the whole island. District boundaries were acquired from the Statistics Institute of French Polynesia. The 29 districts that make up the main island (excluding the 5 islets) define fairly homogeneous geographical units based on the number of households and inhabitants. In these districts the average population is 205 inhabitants (range 131–368, median 200). The island’s main economic activities are vanilla cultivation, copra production, fishing, and tourism. The central, uninhabited, hilly part of the island is covered by dense tropical vegetation. The villages are mainly rural, with dwellings located along the coast. The annual weather cycle is characterized by heavy rainfall during the hot season (November to April, with an average rainfall of 193 mm/month) and lower rainfall during the rest of the year [39]. These seasonal fluctuations are often influenced by the El Niño Southern Oscillation and the lesser-known Madden-Julian (MJO) oscillation, which together affect wind patterns (trade winds), sea temperature and precipitations [40].

### Mosquito sampling and identification

The study was conducted in 2021 at the end of the hot season (01–31 March). Due to logistical constraints, 6 of the 34 districts of Huahine were not included in the study: the 5 islets and one of the smaller, less populated districts. The 28 districts investigated represent our Primary Sampling Units (PSUs) (Fig 1b). In each of the 28 PSUs, 15 households were randomly selected, with a minimum distance of 50 m from each other. In cases where the owner was absent or refused to participate, the next house “on the right as you exit” was sampled. Overall, 420 households were sampled across the island. BG-Pro modular traps with rain shield, powered by 6V power banks (10,000 mAh) and baited with BG-lure attractants (Biogents, GmbH, Regensburg, Germany), were used to collect adult mosquitoes. One trap per household was set outdoors, away from direct sunlight and preferably protected from the rain. Traps were checked once every 24 hours to collect the mosquito bags and replace the batteries. After 48 hours, they were removed from the site and moved to a new PSU. Each day, two PSUs were sampled simultaneously for a total of 30 traps per day.

Mosquitoes were euthanized in a freezer and then sorted, identified, and counted down to species and sex using taxonomic keys [41,42]. Female mosquitoes were pooled (each pool comprising between 1 and 20 specimens) keeping each species, household, and collection date separate, and dry-stored at -20°C before high-throughput analysis at Institut Louis Malardé (ILM, Tahiti, French Polynesia). For laboratory analyses of female mosquitoes other than *Ae. polynesiensis*, the two collection dates were combined (after 24 hours and 48 hours), while keeping the total number of specimens less than or equal to 20. This resulted in a reduction in the number of samples to be analysed, as many collections included only one or a few individuals for these less represented mosquito species. For all species, all female mosquitoes were included in the pools and analysed, regardless of their physiological status (unfed, fed, gravid, etc.).

### Mosquito processing and PCR analyses

DNA was extracted from each mosquito pool using the Chemagic DNA Tissue kit (PerkinElmer chemagen Technologie GmbH, Germany). Pooled mosquitoes were ground in 300 µL of lysis buffer using the Bead Ruptor 96 homogenizer (OMNI International, USA) with 1.5 mm stainless steel metal beads (30 Hz for 5 min). After Proteinase K digestion (56°C for 2 hours, under gentle agitation), DNA extraction was achieved on the Chemagic 360 nucleic acid extractor (PerkinElmer chemagen Technologie GmbH) following the manufacturer’s instructions. PCR were performed as previously described [43] using primers and probes designed to amplify a fragment of the trans-spliced leader RNA gene of *Wuchereria bancrofti*, with slight modifications of the probes. The TaqMan probes were modified as follows: the 5’ dyes were replaced with 6-FAM, and the 3’-terminal quenchers were substituted with BHQ1 to meet the synthesis specifications of the supplier (TIB Molbiol, Berlin, Germany). Internal quenchers were removed without compromising the reaction’s sensitivity. The PCR reaction was run in 1x TaqPath ProAmp Master Mix (Applied Biosystems, Waltham, Massachusetts, USA) in a 25 µL final volume containing 3.1 pM of the forward primer (Wb-CL1-F GCTGAAAATCATTCGCTTTTGAATG), 25 pM of the reverse primer (Wb-CL1-R GGGTAATTAAACCGGTGATCCT), 6.2 pM of probe (Wb-CL1-P 6FAM-ACAACAACTATATGGGAATGGTGCAGGT-BHQ1) and 2.5 µL of mosquito DNA extract. Thermocycling conditions were 50°C for 2 min, 95°C for 10 min, followed by 40 cycles of 95°C for 15 sec and 60°C for 1 min on a CFX96 Touch Real-Time PCR Detection System (BIO-RAD, Hercules, CA, USA). To check for the presence of PCR inhibitors potentially introduced during DNA extraction, an internal amplification control (IAC; 100 pg) was added to each pool prior to extraction. IAC amplification was performed in a 7 µL final volume reaction containing 1.75 pM of the forward primer (IAC-F CTAACCTTCGTGATGAGCAATCG), 1.75 pM of the reverse primer (IAC-R GATCAGCTACGTGAGGTCCTAC), 0.87 pM of probe (IAC-Probe 6FAM-AGCTAGTCGATGCACTCCAGTCCTCCT-BHQ1) and 2 µL of mosquito DNA extract. PCR was performed following the same cycling conditions with a 59°C annealing/elongation step. The *W. bancrofti* positive control was prepared from pools of 10 microfilariae preserved at -80°C in the Institut Louis Malardé laboratory [44]. Filarial DNA was extracted using the DNA easy Blood and tissue kit (Qiagen). IAC positive controls were added on each PCR plate (10, 25 and 50 pg). Two pools of laboratory-reared female mosquitoes were added to each DNA extraction plate: one as a negative control for the PCR run, and the other one spiked with filarial DNA as an additional positive control. Analyses were performed using the CFX manager and Maestro softwares (Bio-RAD). Samples with Cq values ≤ 32 were classified as positive. Those with Cq values > 32 and ≤ 34 were retested, and classified as positive only if amplification was confirmed. Samples with Cq values of zero or > 34 were considered negative, provided the internal control amplified properly (Cq > 17 and ≤ 32).

### Data analysis and visualization

Data on female mosquito collection and PCR analyses were used to create maps showing mosquito abundance and the proportion of PCR-positive pools by species and PSU.

To further understand the extent of *W. bancrofti* infection within the mosquito populations, we estimated infection prevalence using the PoolTestR package [10,45] in RStudio version 4.1.1. This package calculates the proportion of infected mosquitoes from PCR test results on mosquito pools and provides confidence intervals to ensure reliable estimates. The PoolPrev function in PoolTestR uses a maximum likelihood estimation method to determine infection prevalence. This method aims to find the proportion of infected mosquitoes that maximizes the likelihood of observing the actual data collected, thus providing a reliable estimate of infection rates. We utilized the frequentist approach due to the presence of PSUs without LF positive pools. Additionally, we created a graph showing infection prevalence percentages by species, including confidence intervals. The maximum likelihood estimation used in PoolTestR may yield asymmetric confidence intervals that are not centered around the point estimate.

**Fig 2:**
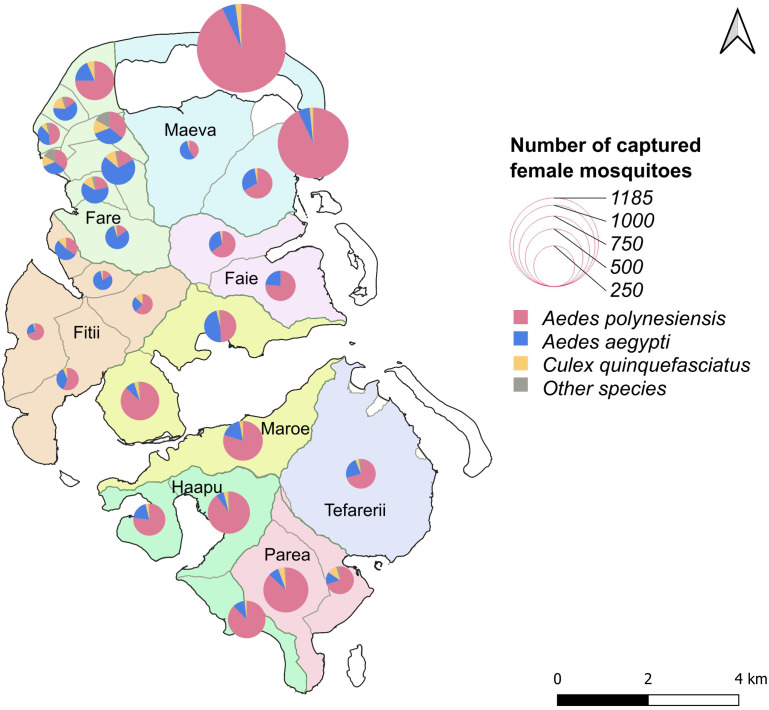
Distribution and abundance of captured female mosquitoes across the sampled PSUs in Huahine. The circles represent the total number of female mosquitoes captured within each PSU; their size is proportional to this number. Colours indicate the proportion of the different species of female mosquitoes *Ae. polynesiensis*, *Ae. aegypti*, and *Cx. quinquefasciatus*. The three least abundant species, *Ae. vexans*, *Cx. annulirostris* and *Tx. amboinensis*, were grouped together under the category “other species”. The base map is sourced from OpenStreetMap contributors (https://www.openstreetmap.org), under the Open Database License (ODbL). The map was generated using QGIS (version 3.30.0, 2023).

**Fig 3:**
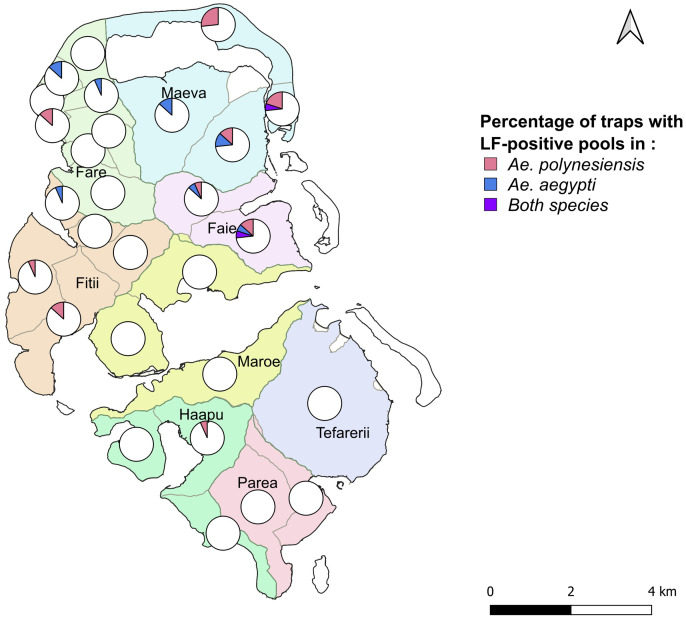
Presence and proportion of traps with *W. bancrofti* PCR-positive mosquito pools by PSU in Huahine. Each circle represents 15 traps (corresponding to 15 households) and the proportion of traps with PCR-positive pools is indicated by different colours, corresponding to the species *Ae. polynesiensis*, *Ae. aegypti* and both species. The base map is sourced from OpenStreetMap contributors (https://www.openstreetmap.org), under the Open Database License (ODbL). The map was generated using QGIS (version 3.30.0, 2023).

**Fig 4:**
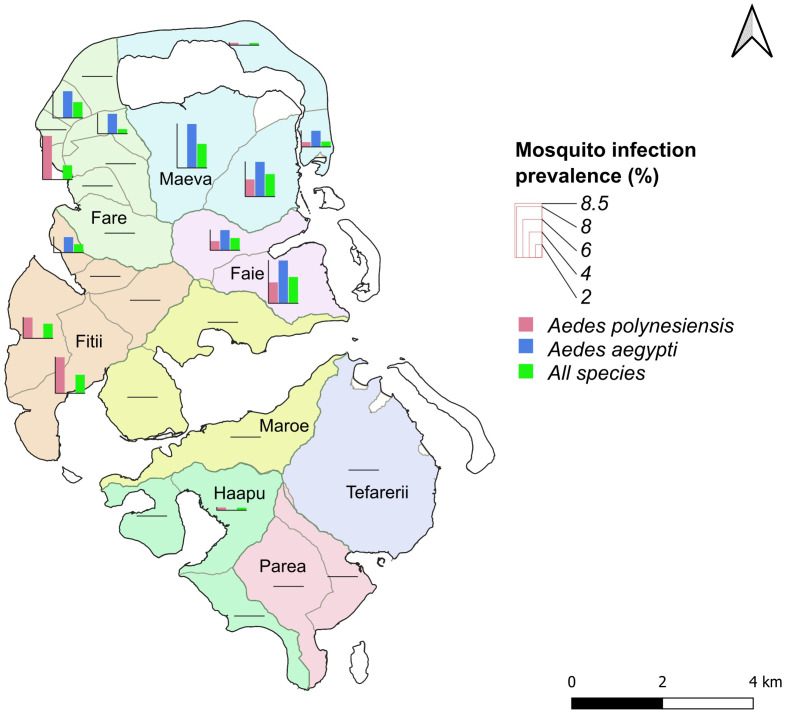
Estimated infection prevalence in mosquitoes by species and PSU in Huahine. Histogram bars represent the estimated infection prevalence (%) for *Ae. polynesiensis*, *Ae. aegypti*, and for all *Aedes* and *Culex* species combined, within each PSU. Prevalence estimates were calculated using the R package PoolTestR. The base map is sourced from OpenStreetMap contributors (https://www.openstreetmap.org), under the Open Database License (ODbL). The map was generated using QGIS (version 3.30.0, 2023).

Maps were created using a background map of Huahine sourced from OpenStreetMap (https://www.openstreetmap.org/#map=12/-16.7501/-150.9806) and the OpenStreetMap Foundation, available under the Open Database License (ODbL, https://opendatacommons.org/licenses/odbl/1-0/) (Figs 1–4). Maps were modified using QGIS software (3.30.0 ‘s-Hertogenbosch, 2023) with the OSMDownloader plugin. Spatial analyses were performed using QGIS software. The QField open-source mobile application (https://qfield.org) was used to acquire household geographical coordinates.

## Results

### Mosquito distribution and abundance

During the campaign, the sampling effort involved 15 traps randomly deployed near human dwellings in each of the 28 PSUs, for a total of 420 collection points. Mosquitoes were collected for two days, at two interval times, after 24 and 48 hours. A total of 5508 female mosquitoes were trapped, averaging 197 specimens per PSU (range 44–1185; median 135) and 13.1 mosquitoes/trap/48 hr period (range 0–260; median 11) (Table 1). Six Culicidae species were identified: *Aedes polynesiensis*, *Aedes aegypti* (Linnaeus, 1762), *Aedes vexans* (Meigen, 1830), *Culex quinquefasciatus* (Say, 1823), *Culex annulirostris* (Skuse, 1889) and *Toxorhynchites amboinensis* (Doleschall, 1857) by order of decreasing abundance. *Ae. polynesiensis* was the predominant species (n = 4062; 73.7%) followed by *Ae. aegypti* (n = 1117; 20.3%), *Cx. quinquefasciatus* (n = 245; 4.4%) and *Ae. vexans* (n = 57; 1.0%). *Cx. annulirostris* (n = 19; 0.4%) and *Tx. amboinensis* (n = 8; 0.2%) were rarely found (Table 1).

**Table 1:** Number of female mosquitoes collected by species in the different PSUs and villages in Huahine.

Village	PSU name	PSU code	*Aedes* *polynesiensis*		*Aedes* *aegypti*		*Culex* *quinquefasciatus*		*Aedes vexans*		*Culex* *annulirostris*		*Toxorhynchites* *amboinensis*		Total (and %)	
**Fare**	Fare North 1	1	172	377	42	466	14	108	1	52	0	6	0	3	229	1012 (18.4)
	Fare North 2	2	13		53		17		1		2		1		87	
	Fare West	3	36		29		6		1		1		1		74	
	Fare East	4	57		53		22		25		1		1		159	
	Fare Center 1	5	33		31		13		16		0		0		93	
	Fare Center 2	6	29		120		19		5		0		0		173	
	Fare Center 3	7	24		69		14		2		2		0		111	
	Fare South	8	13		69		3		1		0		0		86	
**Maeva**	Maeva motu North	9	1101	1916	59	172	24	41	0	1	0	0	1	1	1185	2131 (38.7)
	Maeva motu South	10	700		41		11		1		0		0		753	
	Maeva West	11	22		30		2		0		0		0		54	
	Maeva East	12	93		42		4		0		0		0		139	
**Fitii**	Fitii North 1	13	26	147	41	141	9	24	0	2	0	0	0	0	76	314 (5.7)
	Fitii North 2	14	9		45		2		0		0		0		56	
	Fitii West	15	31		11		1		1		0		0		44	
	Fitii East	16	39		16		8		0		0		0		63	
	Fitii South	17	42		28		4		1		0		0		75	
**Faie**	Faie North	18	69	174	34	67	3	3	0	0	0	0	0	0	106	244 (4.4)
	Faie South	19	105		33		0		0		0		0		138	
**Maroe**	Maroe West	20	195	461	18	132	8	19	0	0	1	3	2	2	224	617 (11.2)
	Maroe North	21	77		73		4		0		2		0		156	
	Maroe South	22	189		41		7		0		0		0		237	
**Haapu**	Haapu West	23	119	546	30	68	5	18	0	0	1	2	0	1	155	635 (11.5)
	Haapu East	24	241		16		9		0		1		1		268	
	Haapu South	25	186		22		4		0		0		0		212	
**Tefarerii**	Tefarerii	26	94	94	31	31	5	5	2	2	0	0	1	1	133	133 (2.4)
**Parea**	Parea West	27	265	347	23	40	14	27	0	0	3	8	0	0	305	422 (7,7)
	Parea East	28	82		17		13		0		5		0		117	
**Total (and %)**			4062 (73.7)		1117 (20.3)		245 (4.4)		57 (1.0)		19 (0.4)		8 (0.2)		5508 (100)	

*Ae. polynesiensis* predominated in most parts of the island, especially in two PSUs in Maeva motu North (n = 1101) and South (n = 700), on the northeast part of the island. Overall, the number of *Ae. polynesiensis* per PSU ranged from 9 to 1101 (mean 145; median 73). *Ae. aegypti* was less abundant, with numbers ranging from 11 to 120 (mean 40; median 33.5), except in four PSUs in Fare, two PSUs in Fitii and one PSU in Maeva (Table 1 and Fig 2).

### Detection and prevalence of LF-positive mosquitoes

The non-haematophagous species *Tx. amboinensis* (8 individuals) was not included in the analyses. The 5500 female mosquitoes were sorted into a total of 1073 PCR pools (mean 5.1 mosquitoes per pool, range 1–20), comprising 611 pools of *Ae. polynesiensis* (mean 6.64 mosquitoes per pool, range 1–20), 298 pools of *Ae. aegypti* (mean 3.69 mosquitoes per pool, range 1–20), 124 pools of *Cx. quinquefasciatus* (mean 1.93 mosquitoes per pool, range 1–10), 27 pools of *Ae. vexans* (mean 2.03 mosquitoes per pool, range 1–8) and 13 pools of *Cx. annulir*ostris (1.31 mosquitoes per pool, range 1–3) (Table 2).

**Table 2:** Detection of *W. bancrofti* DNA in mosquito pools through PCR analysis.

Species	*Aedes polynesiensis*	*Aedes aegypti*	*Culex* *quinquefasciatus*	*Aedes* *vexans*	*Culex annulirostris*	Total
**Number of pools analysed**	611	298	124	27	13	1073
**Percentage per species (%)**	56.9	27.8	11.6	2.5	1.2	100
**Number of positive pools**	24	12	0	0	0	36
**Percentage of positive pools per species (%)**	3.9	4.0	0	0	0	3.4
Number of positive PSUs*	9	8	0	0	0	13**
**Percentage of positive PSUs out of the 28 PSUs (%)**	32.1	28.6	0	0	0	46.4

*A PSU is considered positive if at least one mosquito pool from one trap within the PSU tested PCR-positive **Four of the PSUs were positive for both *Ae. polynesiensis* and *Ae. aegypti*

PCR-positive pools were exclusively from specimens of the two species *Ae. polynesiensis* (66.7%) and *Ae. aegypti* (33.3%). Out of all the pools, 3.4% tested positive for *W. bancrofti* DNA by PCR. More precisely, 3.9% of *Ae. polynesiensis* pools (24/611) and 4.0% of *Ae. aegypti* pools (12/298) were positive (Table 2). During this sampling campaign 46.4% (13/28) of PSUs had at least one PCR-positive pool. PCR-positive pools were from specimens of *Ae. polynesiensis* in 9/28 PSUs (32.1%) and *Ae. aegypti* in 8/28 PSUs (28.6%) (Table 2). Four PSUs had PCR-positive pools from both *Aedes* species. All positive pools were detected in mosquitoes from the northern part of the island, except for one pool in the Haapu East PSU (Fig 3). In total, positive pools were found in 30 households, four of which had *Ae. polynesiensis* mosquitoes from both trap collections (24 and 48 h) testing positive, and two of which had PCR-positive pools for both *Aedes* species.

PoolTestR provided an estimation of the LF infection prevalence in the mosquito species in Huahine. Fig 4 illustrates the spatial variability in infection prevalence among mosquito species, providing insights into the geographic distribution of the infection across the island. The estimated infection prevalence in all species was 0.62% (95% confidence interval [Cl] 0.43-0.85), with the highest prevalence found in *Ae. aegypti* (1.1% [CI] 0.60-1.9), followed by *Ae. polynesiensis* (0.53% [Cl] 0.34-0.79). The highest prevalence in *Ae. aegypti* was found in the villages of Maeva (“Maeva West (11) “(6.91% [CI] 1.18-19.9); “Maeva East (12)” (5.46% [CI] 0.925-16.1)) and Faie (“Faie South (19) “(6.73% [CI] 1.15-19.5)) (Figs 4, S1). The highest prevalence in *Ae. polynesiensis* was in the villages of Fare (“Fare Center 1 (5)” (6.87% [CI] 1.17-20.0)) and Fitii (“Fitii West (15)” (3.28% [CI] 0.190-13.7); “Fitii South (17)” (6.87% [CI] 1.17-20.0)) (Figs 4, S1). All confidence intervals are displayed in S1 Fig. This figure illustrates substantial variability in prevalence estimates across sites, with large standard errors that fluctuate according to both the number of pools tested and the number of mosquitoes per pool. Sites with smaller sample sizes tend to exhibit greater uncertainty, as reflected by wider error bars. Notably, some sites report an estimated prevalence of 0%, yet still display a non-zero standard error, underscoring the statistical uncertainty inherent to sampling. In such cases, the absence of detected positive pools does not definitively indicate zero prevalence. These results should therefore be interpreted with caution: the prevalence estimates primarily provide an indication of infection levels rather than exact values and should not be overinterpreted given their sensitivity to sample size and sampling conditions.

## Discussion

This study focused on investigating the abundance and distribution of mosquito vectors, and on mapping potential LF transmission foci on Huahine Island using a molecular xenomonitoring approach. Our results show that *Ae. polynesiensis*, the primary LF vector in French Polynesia, was the dominant mosquito species in Huahine. Its widespread distribution and abundance across the island, particularly in Maeva village (in the Northeast), likely results from the diverse range of natural larval containers. Indeed, tree holes, coconut shells and terrestrial crab burrows provide the ideal breeding spots for this species. The structure of the Huahine island landscape, particularly in some areas such as the motu Maeva where seaside coconut groves and crabs are abundant, provides such breeding sites. *Ae. aegypti* was also well represented, even surpassing the number of *Ae. polynesiensis* in Fare, the island’s most densely populated village, in line with its bio-ecology and known human host preference. The presence and abundance of these two mosquito vector species highlight a potentially high risk of infectious disease transmission (LF and arboviruses) on the island of Huahine. *Cx. quinquefasciatus*, *Cx. annulirostris* and *Ae. vexans* were also present albeit in lower proportions.

The sampling covered the vast majority of the island’s inhabited areas. The entomological survey spanned four weeks. The local environment around the traps, including factors such as the presence of hosts, breeding sites, and vegetation, may have affected both the number of mosquitoes captured and the PCR-positive pools [46]. For example, Lenin et al. (2022) found that seroprevalence of LF infection markers in Samoa was associated with topographical environmental variables. To better understand these dynamics, further studies are needed to identify potential demographic and environmental factors that influence mosquito and parasite development. Such research could significantly enhance spatial predictions of lymphatic filariasis (LF) risk [12,47,48].

We detected several positive pools for *W. bancrofti* in both *Ae. polynesiensis* (24 positive pools, 3.9%) and *Ae. aegypti* (12 positive pools, 4%) through PCR analyses. *Ae. polynesiensis* PCR-positive pools clearly show that the parasite is circulating in the mosquito vector population, enabling us to locate potential foci of LF transmission.

The proportion of positive pools of *Ae aegypti* mosquitoes was significant indicating that surveying *Ae. aegypti* females in addition to *Ae. polynesiensis* can provide useful LF mapping data. *Ae. aegypti* is not considered a competent vector for LF since microfilariae migrate to the thorax but do not mature to the third larval (L3) infective stage as supported by numerous studies [49–51] including in French Polynesia [52]. One study reported development to the L3 stage in a specific insectary strain of *Ae. aegypti* (Liverpool strain) [53]. Galliard (1947) reported a successful attempt at larval maturation after numerous trials, in *Ae. aegypti* from Puerto Rico, but not from Samoa [54]. Albuquerque (1999) suggested intrinsic differences in the relationship between *W. bancrofti* and its vector, highlighting the need for studies specific to each endemic geographic region [49]. However, *Ae. aegypti* females which primarily bite humans during the day can turn PCR positive after a bloodmeal on an LF infected person. Research on vector competence of *Ae. aegypti* has provided information on the survival of the parasite in this mosquito. The parasite persists long enough after blood digestion in the mosquito (12–14 days) [49,51] to be detected by a PCR approach on trapped mosquitoes [49]. Thus, both *Aedes* species were found to be informative for this MX survey.

All other mosquito species, including the nocturnal *Culex* spp., tested negative for LF. Previous MX studies conducted in Pacific Island countries (Samoa and American Samoa) indicated low LF positivity in *Cx. quinquefasciatus* pools and low estimated prevalence in *Culex* mosquitoes compared to *Aedes* species [10,25,55]. This is likely due to the diurnally sub-periodic nature of the *W. bancrofti* var. *pacifica* strain circulating in French Polynesia and the South Pacific region [6,56,57]. Microfilariae remain in the peripheral blood of their host and reach peak density during the day, which likely reduces the opportunity for nocturnal mosquito species such as *Culex* spp. to become infected during blood meals [58,59].

Our PCR results revealed that *W. bancrofti*-positive mosquito pools were primarly located in the northern part of Huahine Island, in the villages of Maeva, Faie, Fare and Fitii (Fig 3). Since the PCR method used in MX detects Mf DNA rather than specifically targeting the L3 stage, MX alone cannot confirm active transmission. More direct assessments of ongoing transmission would require complementary approaches, such as mosquito dissection or reverse transcriptase-PCR (RT-PCR) to detect gene expression specific to L3 larvae [25,60]. Although MX does not provide a direct measure of ongoing LF transmission, it provides an indirect assessment of human infection. Given the limited dispersal of *Aedes* mosquitoes, the detection of the parasite in mosquitoes strongly suggests the presence of LF carriers nearby [25]. Thus, our findings not only indicate the location of possible transmission foci in these areas, but also pinpoint the presence of filarial-infected individuals.

Estimated mosquito infection prevalence was relatively high in both *Ae. polynesiensis* and *Ae. aegypti*, surpassing the MX threshold (<0.1%) used to decide when to stop MDA for *Aedes* species. Surprisingly, the estimated prevalence in *Ae. aegypti* was almost twice as high as that in *Ae. polynesiensis*. This higher prevalence was particularly notable in Maeva and Faie villages. Furthermore, our results highlight that areas of high infection prevalence do not always coincide with areas of highest mosquito abundance or biting pressure. For example, on the motu of Maeva, despite higher infection prevalence in *Ae. aegypti*, *Ae. polynesiensis* was much more abundant. The strong propensity of *Ae. aegypti* to feed on human hosts may account for the highest prevalence in this species compared with *Ae. polynesiensis*, which feeds on other mammals and birds. Thus, this discrepancy may be due to different behaviours, but also to the high difference in mosquito abundance between the two species. This highlights the importance of sampling these two different species with varying behaviour, distribution and abundance.

This work also provided valuable information that could simplify species identification in future investigations. Our results showed that only *Ae. polynesiensis* and *Ae. aegypti*, the predominant *Aedes* species in French Polynesia, were positive for filariae. In contrast, other *Aedes* spp. and all *Culex* spp. mosquitoes tested negative. Therefore, future sorting and identification efforts could potentially be simplified by excluding *Culex* spp. from subsequent analyses. However, it should be acknowledged that the absence of filarial infection in *Culex* mosquitoes in this study does not preclude the possibility of detecting positive specimens under different conditions. Therefore, while the exclusion of *Culex* spp. could facilitate MX, particularly in light of the nocturnal periodicity of *W. bancrofti* in French Polynesia, it should be periodically reassessed in future surveys.

Among the *Aedes* species, *Ae. polynesiensis* and *Ae. aegypti* accounted for 98.6% of the female *Aedes* mosquitoes collected, with *Ae. vexans* being the only other species captured in the traps (57 individuals). The overall diversity of the culicidian fauna in French Polynesia is relatively low compared with that of other Pacific island countries, such as Samoa or American Samoa [10,25], which harbor a greater variety of *Aedes* species. This limited diversity suggests that species-level identification of *Aedes* mosquitoes could be reduced to genus-level identification, saving time and eliminating the need for specialized entomological expertise. In the context of French Polynesia, focusing the MX analyses on pools composed exclusively of *Aedes* specimens may be sufficient to delineate potential transmission foci and to identify households associated with positive cases, as demonstrated in the present study. Nevertheless, it is important to acknowledge that mosquito infection prevalence and the threshold values relevant for assessing transmission interruption, may vary between *Aedes* species. Accordingly, in epidemiological contexts involving a greater diversity of *Aedes* mosquitoes or when precise thresholds are needed to infer transmission cessation and guide elimination strategy, species-specific identification remains essential to ensure accurate interpretation of MX data.

We did not perform any antigen or microfilaraemia survey in the human population, though it was possible to locate patients approximately at PSU or household level. Partial data on LF cases in Huahine were available. A human antigenemia study in 2020 identified 21 cases across 7 districts of Huahine [31], and additional positive human cases have been recorded by local health authorities at the village scale between 2020 and 2023. These locations align closely with the potential transmission foci identified via MX. To complement our MX study, having precise information on the number and locations of positive LF cases would further enhance our understanding of transmission dynamics.

In conclusion, our study highlights that *Ae. polynesiensis* was the predominant mosquito species on Huahine. We demonstrated that MX is highly effective in detecting female mosquitoes positive for LF, allowing us to identify potential transmission foci. Given the limited dispersal range of *Aedes* mosquitoes, this suggests that infected individuals are likely located nearby, enabling more targeted and precise control efforts. Specifically, *Ae. polynesiensis* exhibited the highest number of positive pools, whereas the non-vector species *Ae. aegypti* showed a higher infection prevalence within the tested pools. While sorting mosquitoes by species may not be crucial, categorizing them by genus could simplify the feasibility and improve the efficiency of MX efforts. Our data on mosquito abundance, number of positive pools, and infection prevalence have provided key insights to guide targeted interventions and identify priority areas, particularly those with high mosquito density and elevated infection rates. These findings support more efficient resource allocation, whether through MDA campaigns to reduce human infection rates or through targeted mosquito control interventions, using long-term, integrated approaches including community mobilization (for the removal of larval containers around households), as well as incompatible insect technique, sterile insect technique or a combination of both innovative control strategies. Such targeted approaches not only optimize control efforts but may also improve the sustainability of LF elimination programmes by focusing on the most affected areas.

MX proves to be an essential tool for public health surveys, enabling effective monitoring of lymphatic filariasis before, during, and after mass drug administration (MDA) campaigns. It plays a crucial role in assessing the effectiveness of treatments and confirming whether filariasis transmission has been successfully interrupted. MX can still detect the presence of LF in areas where TAS is negative (<1%). This aligns with findings from Samoa, where MX results indicated a decrease in filariasis transmission from 2018 to 2019 following a round of triple-drug mass administration [10]. However, a recent community study (antigenemia and microfilaremia) conducted in 2023 revealed that a single round of treatment was insufficient to sustain reductions over 4.5 years, suggesting ongoing transmission [61]. In French Polynesia, our data played a crucial role in the decision-making process regarding the resumption of triple-drug therapy treatments on Huahine in 2023 and 2024. Targeted antigenemia testing in the areas of higher transmission risk and transmission foci defined by our study will be performed and MX will be used for post-MDA monitoring in Huahine.

## Supporting information

S1 Fig Estimated infection prevalence of female mosquitoes by primary sampling unit (PSU) in Huahine. Prevalence was estimated using the PoolTestR package and a frequentist approach. Bars represent the estimated prevalence (%) with 95% confidence intervals, for *Ae. polynesiensis* (pink), *Ae. aegypti* (blue), and all species of *Aedes* and *Culex* combined (green).
(TIF)

S1 File PCR data. Results of PCR targeting the trans-spliced leader RNA gene of *Wuchereria bancrofti* (Wb-CL1).
(XLSX)
