# Supplementary figures and images for: Detection of potential transmission foci of lymphatic filariasis using molecular xenomonitoring in Huahine, French Polynesia
Source: PLoS Negl Trop Dis. 2025 Sep 19;19(9):e0013492. doi: 10.1371/journal.pntd.0013492 (PMC12448981; doi:10.1371/journal.pntd.0013492)

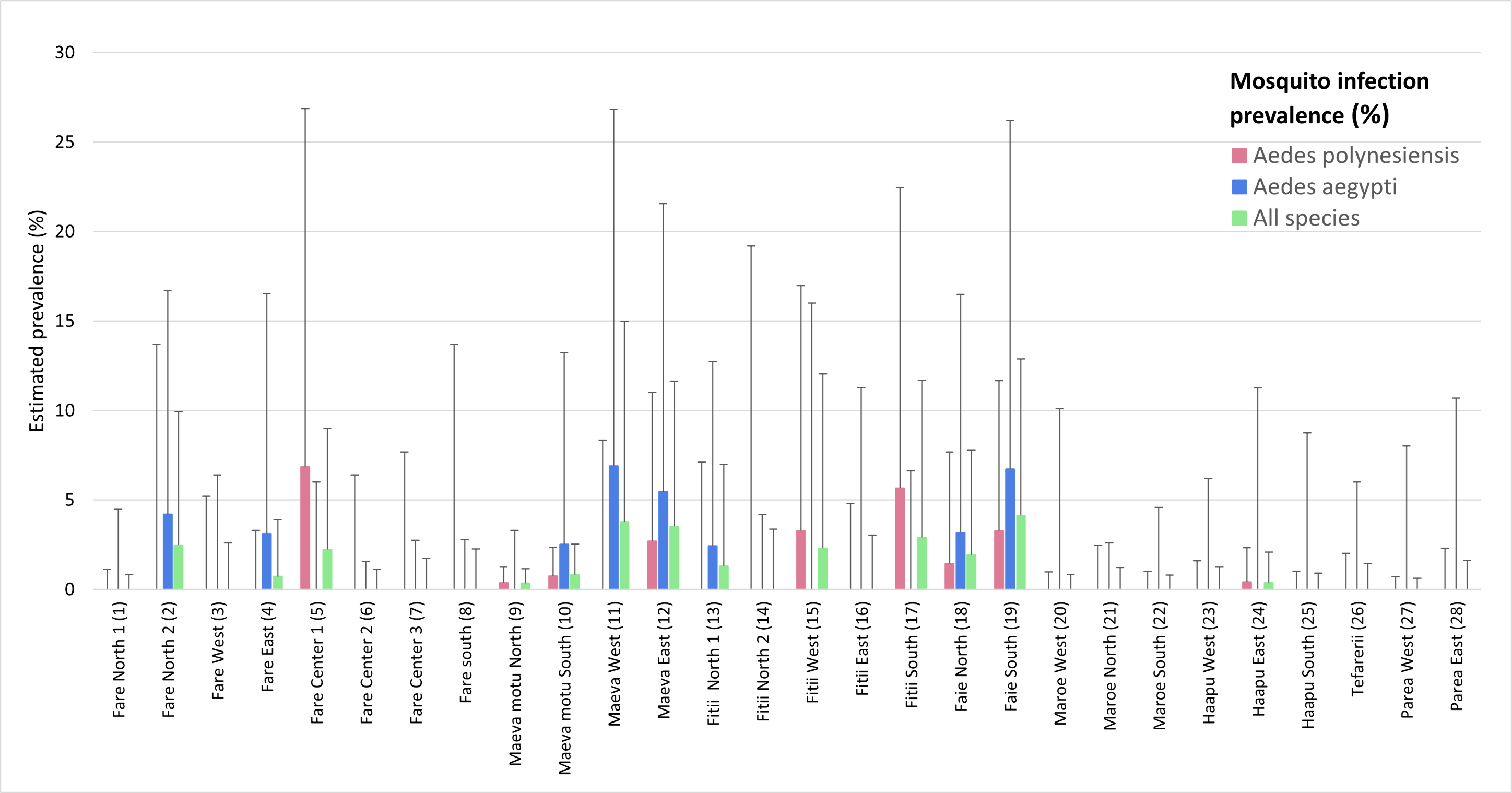

Supplement: S1 Fig — Prevalence was estimated using the PoolTestR package and a frequentist approach. Bars represent the estimated prevalence (%) with 95% confidence intervals, for Ae. polynesiensis (pink), Ae. aegypti (blue), and all species of Aedes and Culex combined (green). (TIF) [file pntd.0013492.s001.tif]
